# Evaluation of image processing technique and discriminant analysis methods in postharvest processing of carrot fruit

**DOI:** 10.1002/fsn3.1614

**Published:** 2020-05-18

**Authors:** Ahmad Jahanbakhshi, Kamran Kheiralipour

**Affiliations:** ^1^ Department of Biosystems Engineering University of Mohaghegh Ardabili Ardabil Iran; ^2^ Department of Biosystems Engineering Ilam University Ilam Iran

**Keywords:** appearance shape, carrot, discriminant analysis, grading, machine vision, waste control

## Abstract

The most important process before packaging and preserving agricultural products is sorting operation. Sort of carrot by human labor is involved in many problems such as high cost and product waste. Image processing is a modern method, which has different applications in agriculture including classification and sorting. The aim of this study was to classify carrot based on shape using image processing technique. For this, 135 samples with different regular and irregular shapes were selected. After image acquisition and preprocessing, some features such as length, width, breadth, perimeter, elongation, compactness, roundness, area, eccentricity, centroid, centroid nonhomogeneity, and width nonhomogeneity were extracted. After feature selection, linear discriminant analysis (LDA) and quadratic discriminant analysis (QDA) methods were used to classify the features. The classification accuracies of the methods were 92.59 and 96.30, respectively. It can be stated that image processing is an effective way in improving the traditional carrot sorting techniques.

## Graphical abstract

Evaluation of image processing technique and discriminant analysis methods in waste control of carrot fruit in postharvest stage.

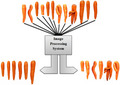

## 1 INTRODUCTION

Carrot (*Daucus carota* L.) is consumed by millions of people all over the world. It belongs to the family Umbelliferae. Carrot is one of the most important and useful vegetables for the human body since it contains nutrients and vitamins. Also, it increases an individual's resistance to infectious diseases (Abbas, 2011; Zhu et al., 2019).

Carrot is used mostly as a raw edible product. One of the carrot problems is shape nonhomogeneity. Although carrots with irregular shapes have no problems regarding their nutritional properties, they are not commonly selected by customers in the markets. This causes to remain the carrots in the markets for long times and then increase the material loss. Therefore, adopting an appropriate method for sorting and packaging this product can increase its desirability in the market and decrease product loss (Jahanbakhshi & Kheiralipour, 2019).

Sorting of agricultural products based on the product quality is one of the most basic and important operations after harvesting. The operation assists customers in recognizing product quality more easily and leads to a more organized distribution and supply of an agricultural product. Until some decades ago, quality control in food industries was carried out by experts. Evidently, in traditional method, the performance is low and it is expensive and inefficient to respond to the increase in consumers' demands. Image processing is a modern technology, which has witnessed considerable progress both theoretically and practically in recent years. The main advantages of using a machine vision system for quality control of agricultural products are the precision and consistency. Recently, the food industry has benefitted from image processing methods and the use of such methods has been successful in nondestructive assessment of the food products (Azarmdel, Mohtasebi, Jafari, & Muñoz, 2019; Jahanbakhshi, Momeny, Mahmoudi, & Zhang, 2020; Kheiralipour & Pormah, 2017; Wang, Sun, Yang, Pu, & Zhu, 2016).

Sorting is defined as putting items into homogenous and uniform classes. This process is one of the most important applications of a machine vision in which objects or products on a line are separated from one another based on their apparent physical properties (Javadikia, Sabzi, & Rabbani, 2017; Mollazade, Omid, & Arefi, 2012; Momin, Yamamoto, Miyamoto, Kondo, & Grift, 2017; Qiaohua, Yihua, & Zhuang, 2017). A fruit's shape is one of the most important criteria and a top priority for quality control by the customers (Fu, Sun, Li, & Wang, 2016; Kheiralipour & Pormah, 2017; Khojastehnazhand, Omid, & Tabatabaeefar, 2010).

Many studies have been conducted in the field of sorting and classifying of products such as kiwi fruit (Fu et al., 2016; Rashidi & Seyfi, 2007), strawberry (Liming & Yanchao, 2010), pear (Zhang & Wu, 2012), tomato (Arjenaki, Moghaddam, & Motlagh, 2013; Clement, Novas, Gázquez, & Manzano‐Agugliaro, 2012), apple (Vivek Venkatesh, Iqbal, Gopal, & Ganesan, 2015), persimmon (Mohammadi, Kheiralipour, & Ghasemi‐Varnamkhasti, 2015), pistachio (Kheiralipour, Ahmadi, Rajabipour, Rafiee, & Javan‐Nikkhah, 2015; Kheiralipour et al., 2016), grapes (Qiaohua et al., 2017), and potato (Al‐Mallahi, Kataoka, Okamoto, & Shibata, 2010; Elmasry, Cubero, Moltó, & Blasco, 2012; Farokhzad, Modaress Motlagh, Ahmadi Moghadam, Jalali Honarmand, & Kheiralipour, 2020) using image processing technique.

Image processing has been vastly applied for fruit and vegetable for detecting size, shape, and defect (Kheiralipour, Ahmadi, Rajabipour, & Rafiee, 2018; Pathmanabana, Gnanavel, & Sundaram Anandan, 2019). Riquelme, Barreiro, Ruiz‐Altisent, and Valero (2008) sorted olive fruits based on the shape of their external defects. First, the fruits were classified into seven categories by experts and then they were categorized according to features such as color and the shape of external defects. Furferi and Carfagni (2010) designed a machine vision system to sort olive fruits based on the ripeness level and external defects. Liming and Yanchao (2010) implemented an automated system for sorting strawberries based on image analysis. The system was able to identify the physical properties of strawberries according to the image features. They reported that the sorting precision was 88.8% based on color features and 90% based on shape features. Mousavi Balestani (2012) discriminated and sorted cherry fruits according to the fruit size, ripeness, and defects using image analysis method. They reported that sorting based on size, ripeness, and defects was carried out with accuracy of 96%, 92%, and 90%, respectively. Elmasry et al. (2012) designed a rapid and accurate machine vision system to distinguish irregular from regular shape potatoes and reported that the practical accuracy of the system was 96.2%. Mohammadi et al. (2015) sorted persimmon fruit based on ripeness level through image processing technique. The results of their study showed that image analysis indicated a significant difference among different ripeness levels for most image features such as R, G, B channels and the gray level. They also reported that quadratic discriminant analysis (QDA) could sort fruits with accuracy of 90.24%. Kheiralipour and Pormah (2017) used image processing technique and artificial neural networks to sort cucumber fruits and reported that the best sorting model was obtained through neural network with the accuracy of 97.1%.

Literature review on the subject shows that there are no reported studies about carrot sorting based on shape. Thus, the aim of the present study was to distinguish the carrot shape using machine vision, which is useful for carrot sorting in order to increase its marketability and waste control of the product.

## 2 MATERIALS AND METHODS

**FIGURE 1:**
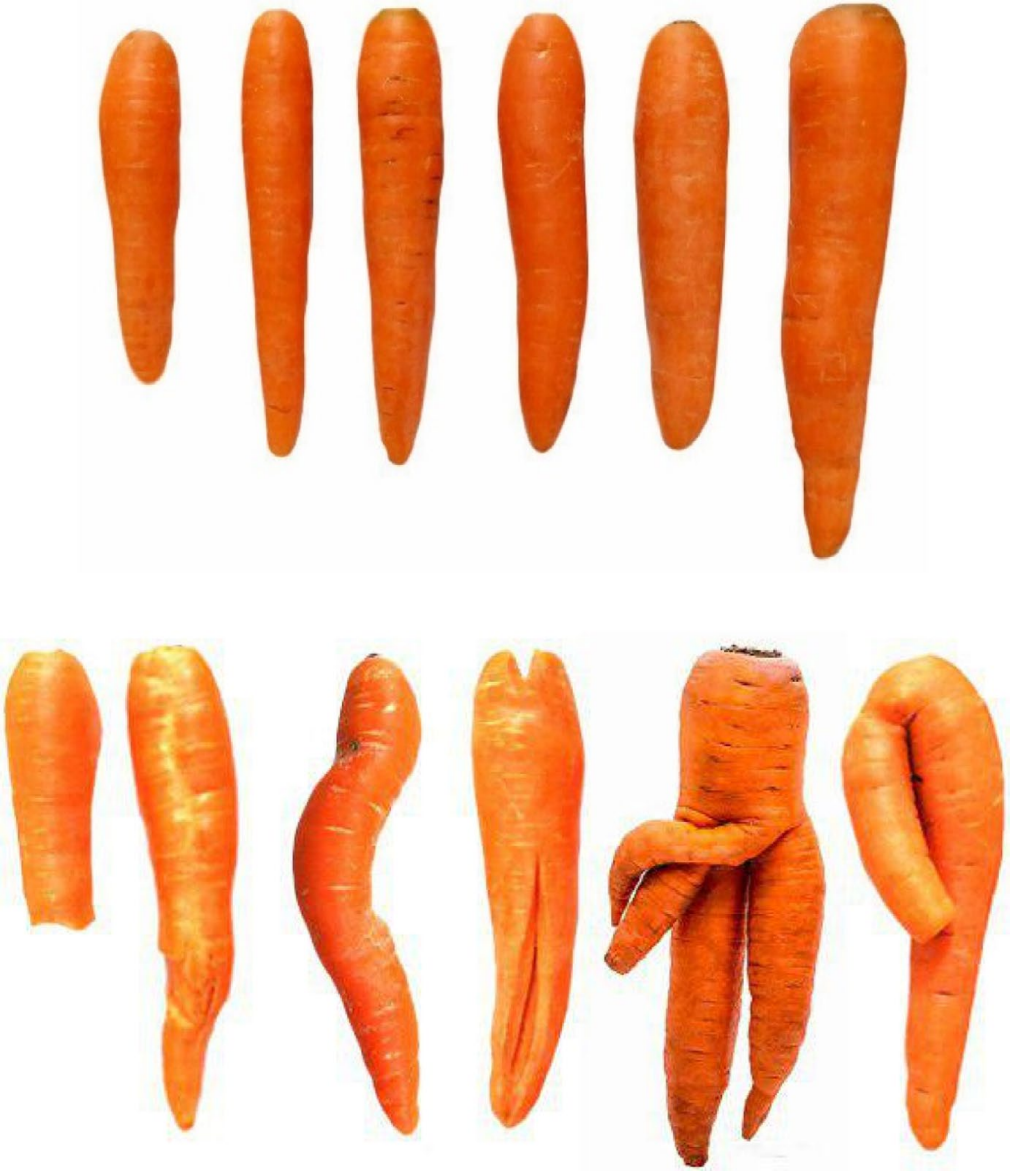
Regular (top) and irregular (bottom) carrot shapes (Jahanbakhshi and Kheiralipour 2019)

In the present study, 135 carrot samples with different shapes (56 regular and 79 irregular) were selected and their images were acquired through an imaging system. First, an expert divided the carrots into two classes: regular and irregular shape. The carrots with irregular shapes were the ones with double or triple roots, curved, damaged, broken, and upright ones (Figure 1).

**FIGURE 2:**
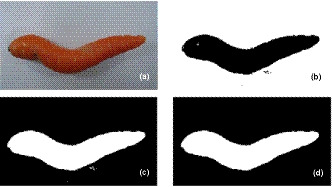
(a) Unchanged carrot image, (b) binary image, (c) reversing the image and filling the hole pixels, and (d) removing the noises

After image acquisition, the obtained images were used for processing by a programmed algorithm in MATLAB R2012a software. The images were first read by the algorithm. Image preprocessing was the first step in processing. In this step, the red, green, and blue channels (R, G, and B) were extracted from the RGB images. Then, the image noises and marginal lines were removed. The blue channel was used for segmentation to separate the carrot from the background so that it could recognize which part of each image channel belongs to the carrot and which part relates to the background (Figure 2a). Thus, carrots in the images were given a dark color (Figure 2b) and the background became bright (Figure 2c). The image holes were eventually filled to complete the carrot shape (Figure 2d).

**FIGURE 3:**
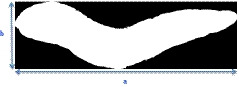
The length (a) and width (b) of carrot image

**FIGURE 4:**
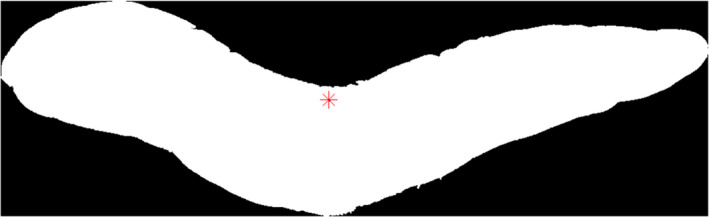
The area center of carrot image

For extraction shape features from the images which are used to distinguish the irregular from regular carrot shape, the image matrix was labeled and length, width (Figure 3), the centroid (Figure 4), area, eccentricity, extent, perimeter, elongation, and the lengths of the large and small axes of the oval around the carrot were calculated.

Other features such as roundness, elongation, and compactness of the carrot images were calculated through Equations (1), (2), (3), respectively:

(1) $$\text{Ro}=\frac{4\pi A}{p^{2}}$$

(2) $$\text{Co}=\frac{p^{2}}{A}$$

(3) $$\text{El}=\frac{a}{b}$$

where Ro is roundness, Co is compactness, El is elongation, *a* is length, *b* is width, *p* is perimeter, and *A* is the area of carrot.

**FIGURE 5:**
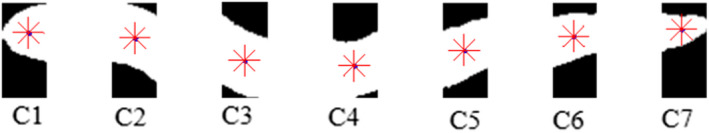
The area center of each carrot image part

Some new features of carrot image were calculated, called partial centroid nonhomogeneity (Fc*_i_*) and partial width nonhomogeneity (Fb*_i_*). For this, the carrot image was divided into seven parts. For calculating partial centroid nonhomogeneity, the centroid of each part of carrot image (c*_i_*) was found (Figure 5).

**FIGURE 6:**
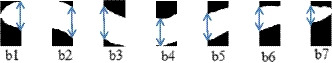
The maximum width of each carrot image part

Then, the centroid of part number 4 was subtracted from that of other parts and six features were obtained as Fc_1_ to Fc_3_ and Fc_5_ to Fc_7_ (Equation 4):

(4) $$\begin{array}{cc} {\text{Fc}}_{i}=c_{i}-c_{4} & i=1-7,i\neq 4 \end{array}$$

where Fc*_i_* is partial centroid nonhomogeneity and c*_i_* is centroid of each image part. The width of each part was also determined to calculate the partial width nonhomogeneity (Fb*_i_*) (Figure 6).

Then, the width of image part number 4 was subtracted from that of other parts and six features were obtained as Fb_1_ to Fb_3_ and Fb_5_ to Fb_7_ (Equation 5):

(5) $$\begin{array}{cc} {\text{Fb}}_{i}=b_{i}-b_{4} & i=1-7,i\neq 4 \end{array}$$

where Fb*_i_* is partial width nonhomogeneity and b*_i_* is the width of each image part.

The total centroid and width nonhomogeneity were extracted (Kheiralipour & Pormah, 2017). The sum of all partial centroid nonhomogeneity was calculated and divided by the biggest carrot width (Equation 6).

(6) $$\begin{array}{cc} {\text{Fc}}_{t}=\frac{\sum \left|{\text{Fc}}_{i}\right|}{\text{bm}} & i=1-7,i\neq 4 \end{array}$$

where Fc_t_ is the total centroid nonhomogeneity, Fc*_i_* is the partial centroid nonhomogeneity of the carrot, and bm is the biggest carrot width. The total width nonhomogeneity was calculated by summing the all partial width nonhomogeneity and dividing by the biggest carrot width (Equation 7).

(7) $$\begin{array}{cc} {\text{Fb}}_{t}=\frac{\sum \left|\text{Fb}i\right|}{\text{bm}} & i=1-7,i\neq 4 \end{array}$$

where Fb_t_ is total width nonhomogeneity, Fb*_i_* is partial width nonhomogeneity, and bm is the largest carrot width (Kheiralipour & Pormah, 2017).

**FIGURE 7:**
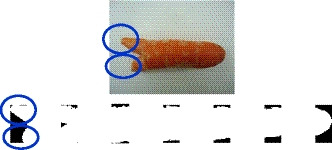
An example of 2‐root carrot

The last feature was extracted as the number of the roots (*N*) of carrot. This feature for single root carrots is equal to 1, but for several root carrots, it is more than 1. In Figure 7, the image of a 2‐root carrot is provided. In this sample, the number of root of the left end part is equal to 2.

In this study, efficient features of carrot shapes were selected for the classification. For this, an algorithm was programmed in MATLAB 2012a software using cross‐validation method based on quadratic discriminant analysis. Then, the carrot images were classified using linear discriminant analysis (LDA) and quadratic discriminant analysis (QDA) using SAS 9.1 software. The efficient features were used as input of the classification methods, and the output was class number, for example, 1 for regular and 2 for irregular shape carrots.

## 3 RESULTS AND DISCUSSION

The efficient features of the carrot sample images were selected using cross‐validation based on quadratic discriminant analysis method. These features that were considered as the input of the LDA and QDA methods are given in Table 1. All features had different values for regular and irregular shapes at 5% probability level.

**TABLE 1:** The efficient features for the classification of carrot

Feature	Mean ± Standard deviation		CV %	
	Regular shape	Irregular shape	Regular shape	Irregular shape
Width	922.859 ± 116.701	1,082.692 ± 155.551	12.64	14.36
Extent	0.757 ± 0.028	0.643 ± 0.061	3.69	9.48
Perimeter	10,507.577 ± 1,098.383	9,797.165 ± 770.783	7.86	10.45
Roundness	897,945.073 ± 219,283.875	1,053,465.075 ± 265,848.023	25.23	24.42
FC2	1.327 ± 0.949	0.335 ± 0.325	97.01	71.51
FC3	1.665 ± 1.122	0.568 ± 0.405	71.30	67.38
FC5	0.503 ± 0.454	1.520 ± 1.239	90.25	81.17
FC6	0.282 ± 0.212	1.437 ± 0.856	75.17	59.56
FCt	5.624 ± 3.213	16.689 ± 7.446	57.13	44.61
Fb1	15.328 ± 8.513	16.055 ± 10.518	55.53	65.51
Fb2	16.605 ± 9.578	17.073 ± 12.143	57.68	71.12
Fb3	20.128 ± 6.912	21.512 ± 14.148	34.34	56.76
Fb4	21.668 ± 3.239	25.544 ± 8.297	14.94	32.48
Fb5	20.419 ± 8.956	25.295 ± 14.179	43.86	56.05
Fb6	16.091 ± 12.480	19.581 ± 16.557	77.55	84.55
Number of roots	1 ± 0	1.166 ± 0.375	0	32.16

Note

The unit of all features is pixel except for the number of roots.

The average width of regular and irregular carrots was equal to 922.86 and 1,082.69, respectively, and the mean of their perimeter was equal to 9,797.17 and 10,507.58, respectively, which indicates that the average width and perimeter of the irregular carrot were more than those of regular one due to inappropriate shape of the appearance shape of the irregular shaped carrots.

The roundness of the studied groups with average values of 1,053,465.08 and 897,945.07 showed that the roundness of irregular carrots was lower than that of regular shape carrots. Also, the averages of the remained features in Table 1 including Fc_2_ to Fb_6_ and number of roots for regular carrots were lower than those of irregular ones. According to differences between the data of regular and irregular carrot shapes (Table 1), there can be told that the features are useful for the classification of the two groups.

In similar studies, Wang and Nguang (2007), Sabliov, Boldor, Keener, and Farkas (2002), Koc (2007) and Omid, Khojastehnazhand, and Tabatabaeefar (2010) emphasized on the use of image processing systems as a new nondestructive method for extracting geometric properties of agricultural products for sorting and grading.

**FIGURE 8:**
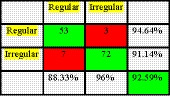
The results obtained by LDA algorithm

The confusion matrix of linear discriminant analysis (LDA) is given in Figure 8. The first category is related to regular shape carrot, and the second one is representative of irregular shape carrots.

As shown in Figure 8, 53 out of 56 samples of regular shape samples have been correctly distinguished by LDA, while three samples have been wrongly identified as irregular shape carrot. From 79 irregular shape carrots, 72 samples have been correctly recognized as irregular shape but seven samples have been wrongly identified as regular shape carrot. In the end, as can be seen in Figure 8, the LDA method could be able to classify carrot samples with correct classification rate of 92.59%.

**FIGURE 9:**
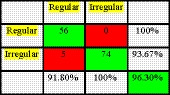
The results obtained by the QDA algorithm

The results of the quadratic discriminant analysis (QDA) method are shown in Figure 9. According to Figure 9, correct classification rate of the QDA method was 96.30%. In this method, all 56 samples of regular shape carrots have been correctly distinguished but five out of 79 irregular shape carrot samples have been wrongly identified.

Image processing technique was applied for shape detection and sorting of products based on shape. Elmasry et al. (2012) sorted potatoes based on their apparent shapes and obtained classification accuracy of 96.2%. Kheiralipour and Pormah (2017) conducted a study to detect desirable and undesirable cucumber shape. They reported 95.7% as correct classification rate of artificial neural network classifier for the classification of cucumber shape. The result of the QDA method (96.3%) in the present study is comparable with similar studies.

The obtained results in the present study indicated the robust ability of the hypothesis to separate regular carrot shapes from irregular ones by image processing technique aligned with the QDA method. Applying sorting machine in this regard besides increasing separating accuracy and decreasing costs, it assists to have significant decrease in product losses. Product loss management by sorting facilities removes the remaining time of products in the markets because of low marketability irregular shape products and allows direct entering of those to processing units such as salad, pickling, and food processing factories.

## 4 CONCLUSIONS

In the present study, carrot shapes were classified into two classes, for example, regular and irregular shapes, according to their physical shapes. After the acquisition of carrot images, some efficient features were obtained and classified by linear discriminant analysis (LDA) and quadratic discriminant analysis (QDA) methods. The results showed that correct classification rates for the methods were 92.59 and 96.30%, respectively. Eventually, it became known that the quadratic discriminant analysis method can sort carrots with high accuracy based on their shapes.

## CONFLICT OF INTEREST

The authors have declared no conflict of interest.
